# Supplementary material for: Comparative Transcriptomic Analysis Reveals the Functionally Segmented Intestine in Tunicate Ascidian
Source: Int J Mol Sci. 2023 Mar 27;24(7):6270. doi: 10.3390/ijms24076270 (PMC10094616; doi:10.3390/ijms24076270)
Supplement: Supplementary file 1 [file ijms-24-06270-s001.zip › 2023-03-02-supplemental-IJMS-zw.pdf]

International Journal of *Molecular Sciences*

MDPI

Article

Comparative Transcriptomic Analysis Reveals the Functionally Segmented Intestine in Tunicate Ascidian

Wei Zhang [1], An Jiang [1], Haiyan Yu [1,*] and Bo Dong [1,2,3,*]

[1] Fang Zongxi Center, MoE Key Laboratory of Marine Genetics and Breeding, College of Marine Life Sciences, Ocean University of China, Qingdao 266003, China
[2] Laoshan Laboratory for Marine Science and Technology, Qingdao 266237, China
[3] MoE Key Laboratory of Evolution and Marine Biodiversity, Ocean University of China, Qingdao 266003, China
* Correspondence: haiyanyu@ouc.edu.cn (H.Y.); bodong@ouc.edu.cn (B.D.); Tel.: +86-0532-82032732 (B.D.)

Citation: Zhang, W.; Jiang, A.; Yu, H.; Dong, B. Comparative Transcriptomic Analysis Reveals the Functionally Segmented Intestine in Tunicate Ascidian. *Int. J. Mol. Sci.* **2023**, *23*, x. https://doi.org/10.3390/xxxxx

Academic Editor: Alfredo Ciccodicola

Received: 3 March 2023
Revised: 16 March 2023
Accepted: 25 March 2023
Published: 27 March 2023

Copyright: © 2023 by the authors. Submitted for possible open access publication under the terms and conditions of the Creative Commons Attribution (CC BY) license (https://creativecommons.org/licenses/by/4.0/).

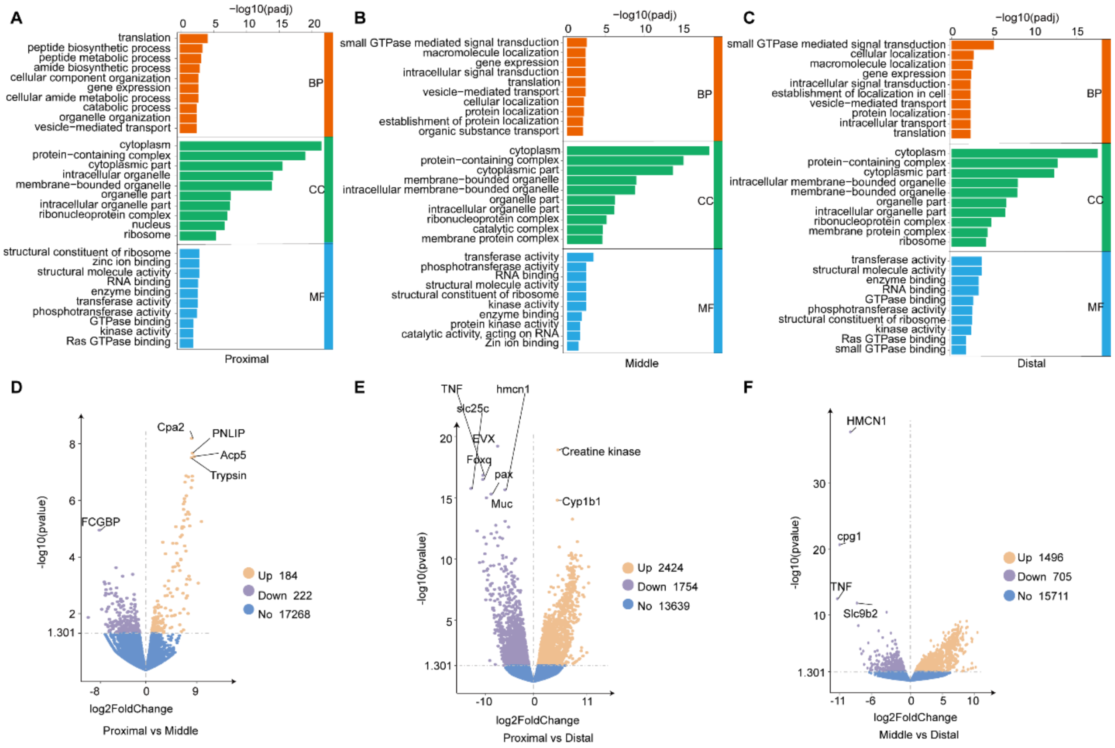

Figure S1: GO enrichment analysis of genes and DEGs enrichment analysis in proximal, middle and distal. (A) All genes of proximal intestine enrichment in Biological Process (BP), Cellular Component (CC) and Molecular Function (MF) terms. (B) All genes of middle intestine enrichment in Biological Process (BP), Cellular Component (CC) and Molecular Function (MF) terms. (C) All genes of distal intestine enrichment in Biological Process (BP), Cellular Component (CC) and Molecular Function (MF) terms. (D) Differential expression analysis of digestive segment compared with absorption segment in log2FoldChange ≥ 2 representing promising candidates. (E) Differential expression analysis of digestive segment compared with immune segment in log2FoldChange ≥ 2 representing promising candidates. (F) Differential expression analysis of absorption segment compared with immune segment in log2FoldChange≥2 representing promising candidates.

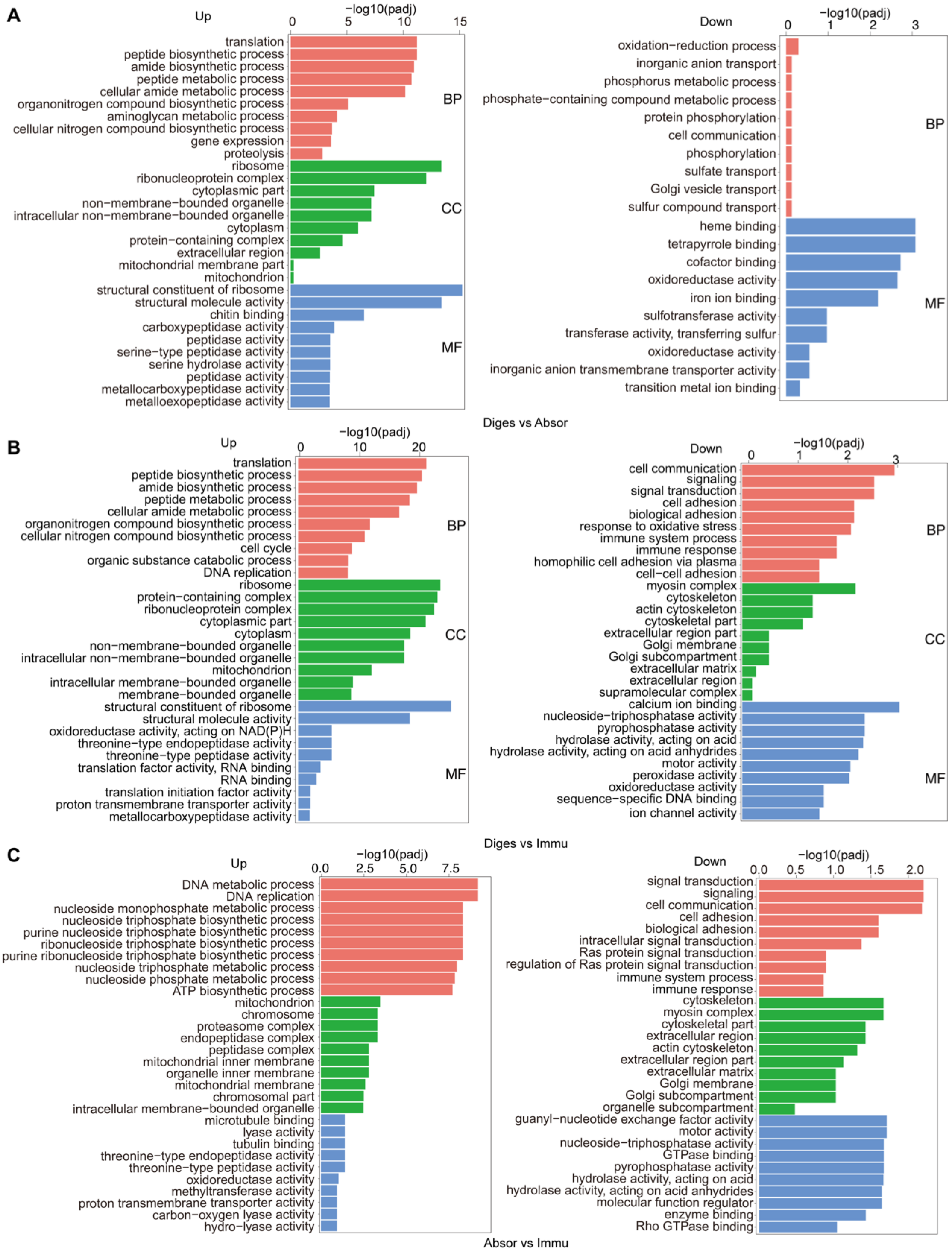

Figure S2: GO enrichment analysis of DEGs. (A) GO enrichment analysis for up-regulated genes and down-regulated genes in Diges vs Absor. (B) GO enrichment analysis for up-regulated genes and down-regulated genes in Diges vs Immu. (C) GO enrichment analysis for up-regulated genes and down-regulated genes in Absor vs Immu. (Diges: Digestive segment, Absor: Absorption segment, Immu: Immune segment).

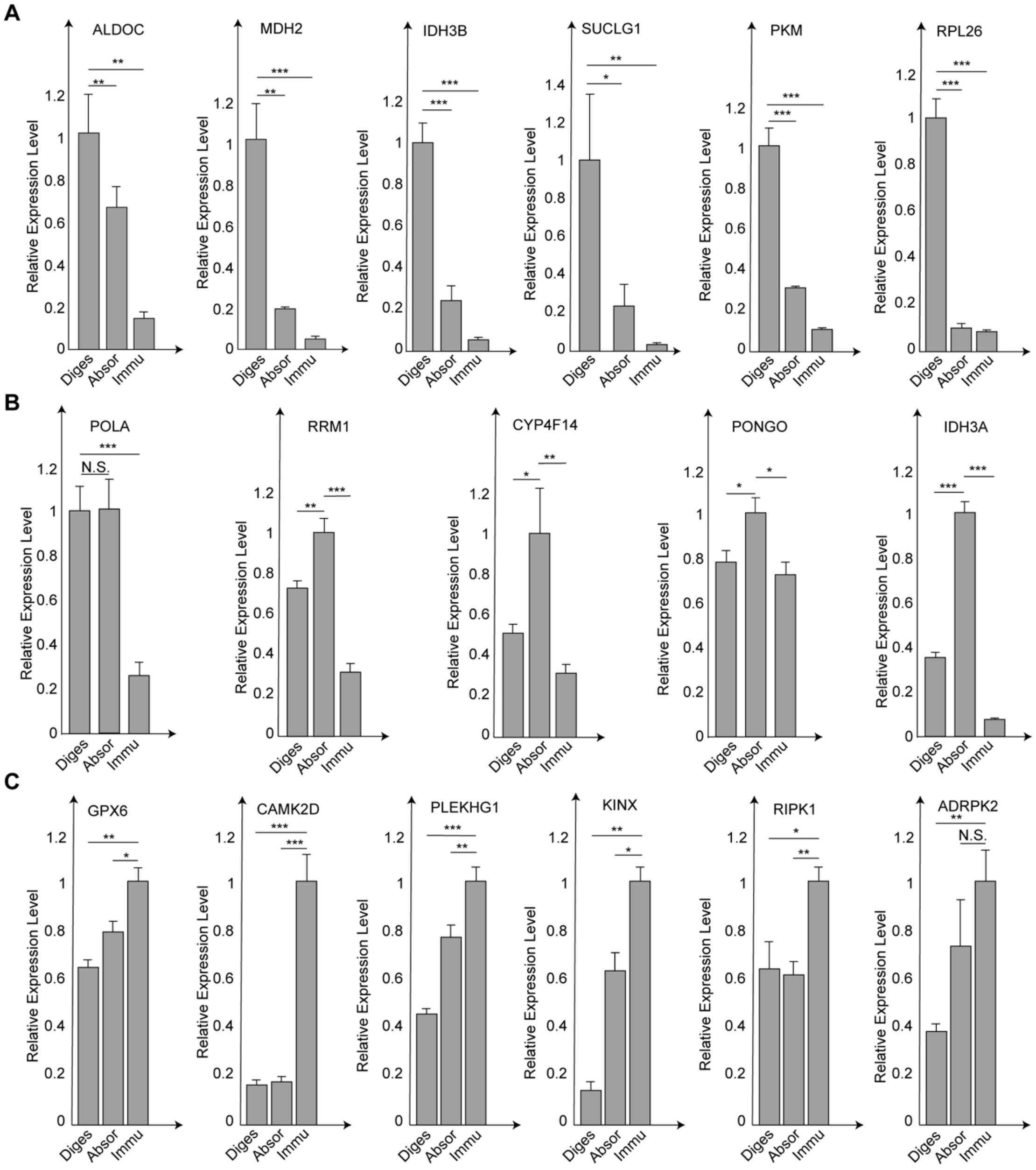

Figure S3: Validation of the RNA-Seq data by qPCR. (A) The expression of DEGs (releated to digestion) in digestive segment was higher than absorption segment and immune segment. (B) The expression of DEGs in absorption segment (releted to absorption) was higher than digestive segment and immune segment. (C) The expression of DEGs (related to immune) in immune segment was higher than digestive segment and absorption segment. Error bars represent standard deviation. ***p < 0.001, **p < 0.01 and *p < 0.05 by Students t-test. (Diges: Digestive segment, Absor: Absorption segment, Immu: Immune segment).

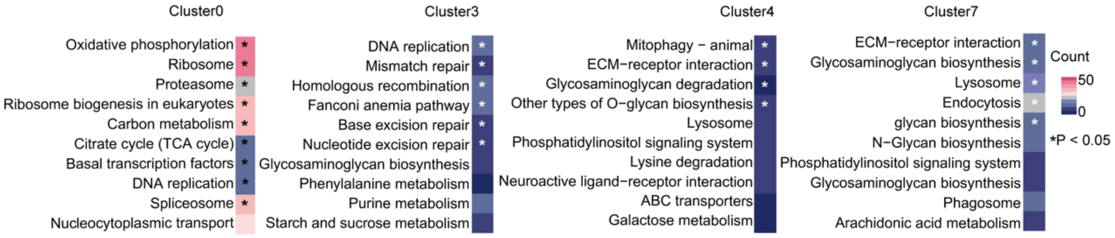

Figure S4: KEGG enrichment in cluster0, cluster3, cluster4, cluster7. The color indicates the gene count, * indicates the p-value < 0.05.

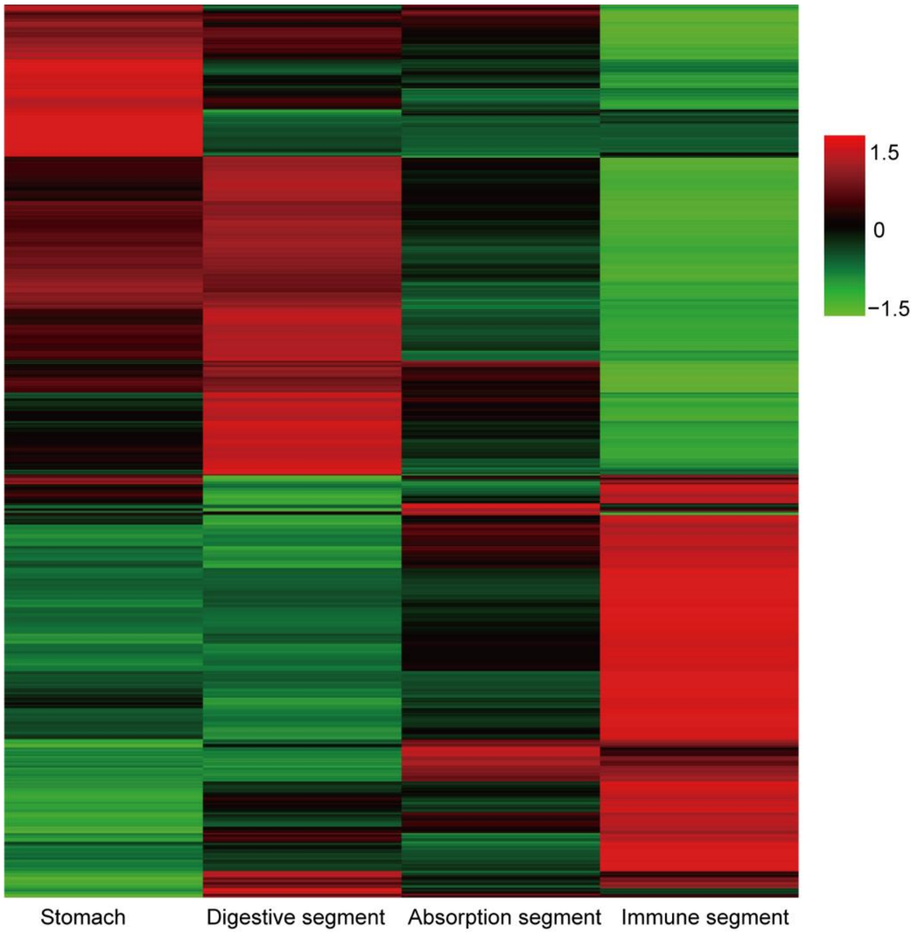

Figure S5: Heatmap displaying Z-score transformed expression of digestive segment, absorption segment, immune segment and stomach. The row Z-score was calculated by log_2-transformed FPKM values of targeted genes. The formula to calculate row Z-scores from FPKM values was $log_2(FPKM+1)$. The row Z-score value ranges from -1.5 to 1.5 and is represented as a color-coded box, with red and green indicating relative up-regulation and down-regulation, respectively.

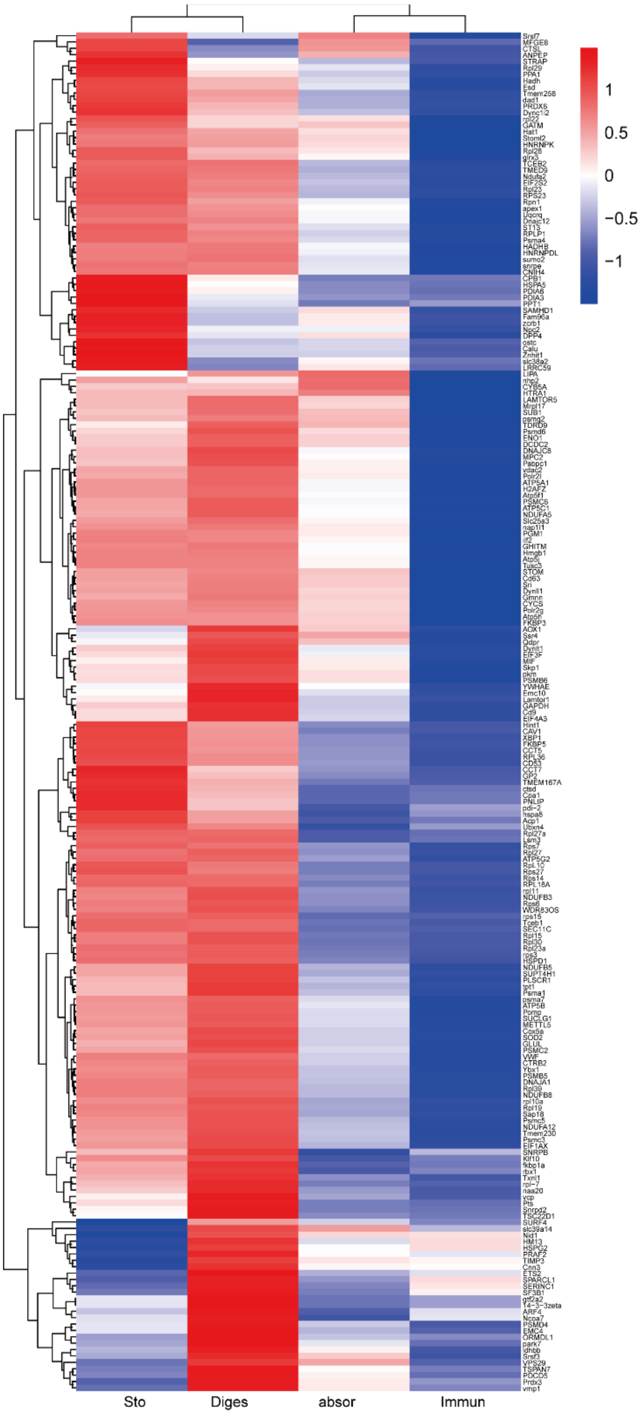

Figure S6: Heatmap of expression of homolougs of Mus musculus pancreatic marker genes in C. savignyi stomach and different intestinal segments**.** (sto: stomach; Diges: Digestive segment; Absor: Absorption segment; Immu: Immune segment).

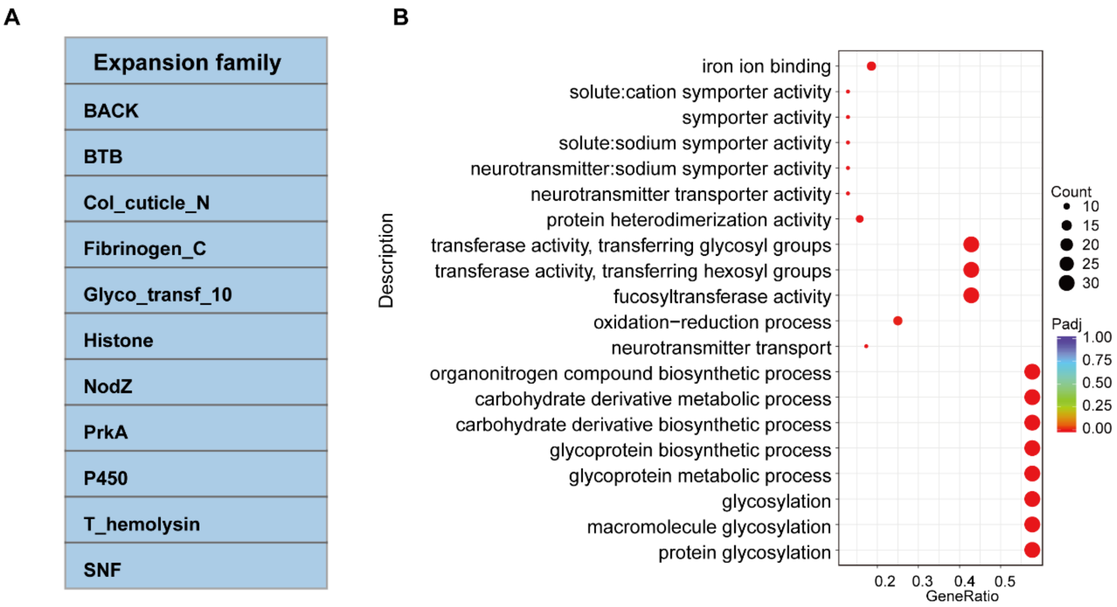

Figure S7: The expanded gene families in *C. savignyi*. **(**A) The representative expanded gene families in *C. savignyi*. (B) GO enrichment analysis of expanded family genes in *C. savignyi*.

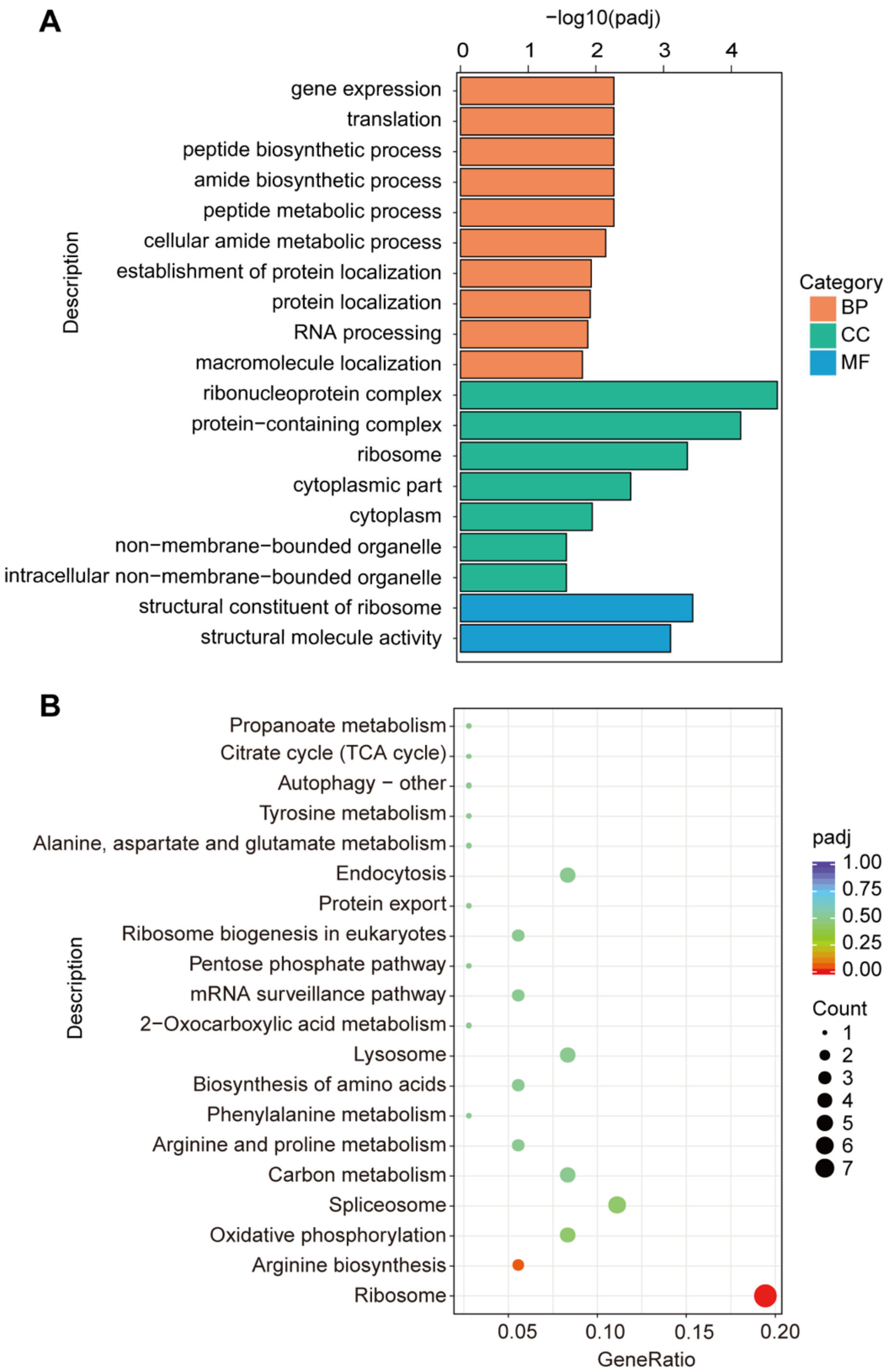

Figure S7: Single-copy genes functional enrichment analysis. (A) GO enrichment analysis of single copy orthologous genes in cross-species. (B) KEGG enrichment analysis of single copy orthologous genes in cross-species.
